# Plasma Protein Biomarkers to Detect Early Gastric Preneoplasia and Cancer: A Prospective Study

**DOI:** 10.3390/ijms262010114

**Published:** 2025-10-17

**Authors:** Quentin Giai Gianetto, Valérie Michel, Thibaut Douché, Karine Nozeret, Aziz Zaanan, Oriane Colussi, Isabelle Trouilloud, Simon Pernot, Marie-Noelle Ungeheuer, Catherine Julié, Nathalie Jolly, Julien Taïeb, Dominique Lamarque, Mariette Matondo, Eliette Touati

**Affiliations:** 1 Institut Pasteur, Université Paris Cité, UAR CNRS 2024, Proteomics Core Facility, MSBio, 75015 Paris, France; quentin.giaigianetto@pasteur.fr (Q.G.G.); thibaut.douche@pasteur.fr (T.D.); mariette.matondo@pasteur.fr (M.M.); 2 Institut Pasteur, Université Paris Cité, Bioinformatics and Biostatistics Hub, 75015 Paris, France; 3 Institut Pasteur, Université Paris Cité, CNRS UMR 6047, Infection, Genotoxicity and Cancer Team, 75015 Paris, France; valerie.michel@pasteur.fr (V.M.); karine.nozeret@pasteur.fr (K.N.); 4 Assitance Publique-Hôpitaux de Paris, Université Paris-Cité, Georges Pompidou European Hospital, Department of Gastroenterology and Digestive Oncology, 75015 Paris, France; aziz.zaanan@aphp.fr (A.Z.); ocolussi@gmail.com (O.C.); isabelle.trouilloud@aphp.fr (I.T.); s.pernot@bordeaux.unicancer.fr (S.P.); julien.taieb@aphp.fr (J.T.); 5 Institut Pasteur, Center for Translational Research, Clinical Investigation and Access to Clinical Resources (ICAReB), 75015 Paris, France; 6 Assistance Publique-Hôpitaux de Paris, CHU Paris IdF Ouest, Pathology Unit Ambroise Paré Hospital, 92100 Boulogne Billancourt, France; catherine.julie@aphp.fr; 7 Institut Pasteur, Université Paris Cité, Clinical Research Coordination Office, 75015 Paris, France; 8 Université Paris-Cité, Department of Gastroenterology and Digestive Oncology, Georges Pompidou European Hospital, SIRIC CARPEM, 75015 Paris, France; 9 Assistance Publique-Hôpitaux de Paris CHU Paris IdF Ouest, Unit of Hepato-Gastroenterology, Ambroise Paré Hospital, 92100 Boulogne-Billancourt, France; lamarquedominique@gmail.com

**Keywords:** liquid biopsy, diagnosis, intestinal metaplasia, dysplasia, gastric cancer, mass spectrometry-based proteomic, early detection, preventive strategies

## Abstract

Gastric cancer (GC) often presents a poor prognosis due to its asymptomatic phenotype at early stages. Upper endoscopy, which is the current gold standard to diagnose GC, is invasive with limited sensitivity for detecting gastric preneoplasia. Non-invasive biomarkers, such as blood circulating proteins, offer a promising alternative for the early detection of gastric lesions. In this prospective study, we identified plasma protein biomarkers for gastric preneoplasia and cancer using mass spectrometry-based proteomics in an exploratory cohort (n = 39). Fifteen promising protein candidates emerged to distinguish patient categories and were further confirmed by enzyme-linked immunosorbent assays (ELISA) in plasma samples from a validation cohort of 138 participants. Our predictive models demonstrated high classification performance with a minimal set of biomarkers. A four-protein panel (ARG1, CA2, F13A1, S100A12) achieved 94.1–98.2% AUROC (95% CI) for distinguishing cancer from non-cancer cases, while a five-protein panel (ARG1, CA2, HPT, MAN2A1, LBP) reached 97.3–99.5% AUROC (95% CI) for distinguishing cancer or preneoplasia from healthy or non-atrophic gastritis cases on the full cohort. Leveraging simple blood sampling, this strategy holds promise to detect high-risk gastric lesions, even at asymptomatic stages. Such an approach could significantly improve early detection and clinical management of GC, offering direct benefit for patients.

## 1. Introduction

Gastric cancer (GC) is the fifth-most commonly diagnosed cancer and the fourth leading cause of cancer-related death worldwide [1]. With over 1 million new cases reported annually, GC remains an important healthcare challenge. It predominantly affects individuals over the age of 60, preferentially men, and it is strongly associated with *H. pylori* infection [2]. *H. pylori* infects nearly half of the global population and is recognized as a major risk factor for GC [3]. While the global incidence and mortality of GC have declined over the past decades, a concerning rise in cases among younger individuals (<50 years) has been observed in both low- and high-income countries [4,5]. Due to its asymptomatic phenotype during the first steps of its development, GC is mainly associated with a poor prognosis, highlighting the importance of its early detection. Effective early detection strategies are particularly critical with important benefits for patient outcomes, as observed in countries with established screening programs [6]. The incidence of GC is higher in Eastern Asia, as in Japan and South Korea, where national screening programs have been implemented leading to a five-year survival of about 60%, compared to 15–20% in Western countries, thus highlighting the benefit of prevention [7].

The majority of GC cases are adenocarcinoma that develops through a multistep process, initiated by a chronic inflammation and progressing through non-atrophic gastritis (NAG) also referred to as gastritis, atrophic gastritis (AG), intestinal metaplasia (IM), and dysplasia (DYS) preceding cancer lesions [8]. If detected at an early stage, meaning AG and/or low-grade preneoplasia, GC can be a preventable disease [9], making the identification of these early-stage lesions a key clinical priority. The prevalence of *H. pylori*-associated AG and IM increases logarithmically with age and confers a higher risk of GC [10]. While *H. pylori* eradication can reduce the risk of GC, residual risk persists, especially in individuals with existing gastric preneoplasia or high baseline risk [11,12]. Presently, upper endoscopy is the gold standard for GC diagnosis and preneoplasia monitoring [13,14]. However, this invasive method is costly and often limited in detecting transient lesions such as DYS [13,14]. It has been reported that 10–20% of patients with GC had no preneoplastic lesions detected by endoscopy within 6 to 36 months prior to the diagnosis [15], underscoring the limitations of current diagnostic approaches and the urgent need for complementary detection methods. Therefore, the discovery of liquid biopsy-based biomarkers, as in blood, is of paramount interest to identify the presence of gastric lesions at an asymptomatic stage. There is a real need for this in clinics, not only for screening and early detection of GC but also for patient follow-up and treatment monitoring.

Circulating proteins have emerged as promising biomarkers for cancer detection, as previously reported for the CancerSeek test [16]. Although glycoproteins including carbohydrate antigen 12-5 (CA12-5), 19-9 (CA19-9), and carcinoembryonic antigen (CEA) are routinely used in clinical practice, their sensitivity and specificity for GC remain limited [17]. Particularly for early GC, where these conventional markers show extremely low positive rates, ranging from merely 2.5% to 15.4% of cases associated with elevated marker levels [18]. Serological markers like the well-studied pepsinogen I and II (PGI and PGII) and PGI/PGII ratio show some predictive value for corpus AG with a sensitivity from 67% to 85% and a specificity from 73% to 87% [19,20]. Despite many efforts, none of the reported blood-based GC biomarkers have been translated into clinical practice to date. This is mainly due to their insufficient sensitivity for early-stage lesions, the lack of large-scale validation studies, and the challenges in standardization across different patient populations.

Recent improvements in high-throughput technologies, particularly mass spectrometry-based proteomics, have enabled the identification of biomarker panels with higher diagnostic accuracy compared to single proteins. For instance, a signature of 19 proteins has been identified to distinguish early-stage (I/II) GC from healthy subjects [21]. However, a significant knowledge gap exists due to the scarcity of prospective studies examining preneoplastic lesions, with most research limited to retrospective binary comparisons between cancer and healthy controls [22].

Our study addresses this gap, using liquid chromatography coupled to tandem mass spectrometry (LC-MS/MS) proteomics to identify plasma protein signatures associated with each stage of gastric lesions progression. This approach allows identification of biomarkers across the entire carcinogenesis cascade, particularly at early, reversible stages where intervention could prevent malignant progression. Using LC-MS/MS for discovery followed by ELISA validation in a larger cohort, we developed clinically applicable predictive models. This comprehensive approach offers several advantages over previous studies: it examines multiple disease stages rather than binary comparisons, employs rigorous validation in a substantial patient cohort and prioritizes biomarkers with practical clinical utility. Our findings provide novel insights into protein biomarker panels that could significantly improve risk stratification and early detection for individuals at risk of GC, potentially transforming the clinical management of this deadly disease.

## 2. Results

### 2.1. Characteristics of the Studied Cohort and Patient Diagnosis

The general characteristics of the patients included in the cohort are reported in Table 1. All patients were previously diagnosed with upper gastrointestinal (GI) endoscopy, and those with AG had undergone upper GI endoscopy within 12 months prior to inclusion. Endoscopic examination of the mucosa was performed under white and blue light, in order to obtain biopsies from sites with the most severe lesions of atrophy or metaplasia, in accordance with European recommendations [13]. Eighteen biopsy samples were systematically taken from the four sides of the antrum, angulus, and the gastric body. The group of preneoplasia is heterogenous and includes patients with AG (21%), IM (47%), and both IM and DYS (32%). 58%, 61%, and 28% of patients with NAG, preneoplasia and GC, respectively, were *H. pylori*-positive. All healthy individuals in the control group are *H. pylori*-negative with mean age significantly lower compared to gastritis, preneoplasia and GC patients (Table 1). Despite the limited number of patients in each group, our study design ensured adequate statistical power and model stability for the multivariate analyses presented (see Section 4.4). Post hoc power analysis demonstrated that our sample sizes provided high power (>98%) to detect large effect sizes (Cohen’s d = 0.8) with two sample *t*-tests in our key comparisons (Figure S1).

### 2.2. Multivariate Analysis of the LC-MS/MS-Based Proteomics Data

**Figure 1:**
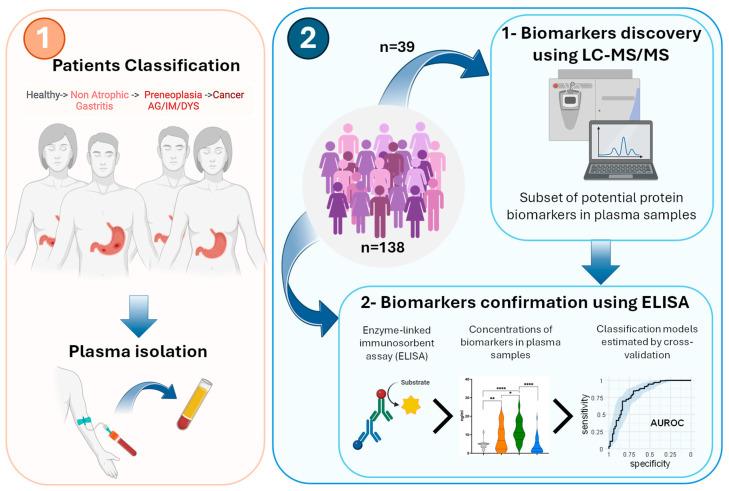
Schematic representation of the study workflow. (**1**) Overview of the setting up of a new multicenter cohort including a group of healthy individuals and three groups of patients with gastritis, preneoplasia and GC (n = 138). The preneoplasia group corresponds to patients with AG with or without IM or DYS. From each patient, a blood sample is collected, and plasma is isolated. (**2**) An exploratory proteomic analysis by LC-MS/MS is performed on plasma samples from a subgroup of patients and healthy subjects (n = 39). The concentrations of potential biomarkers are next measured by ELISA for all participants (n = 138), and their combinations are assessed with prediction models using 4-fold cross-validations repeated ten times. Comparisons of biomarker concentrations were performed using Mann–Whitney tests (**** *p* < 0.0001; ** *p* < 0.01; * *p* < 0.05).

As illustrated in Figure 1, the first exploration phase was conducted using LC-MS/MS-based proteomics on plasma from 39 individuals of the cohort, including healthy patients and patients diagnosed with gastritis, preneoplasia, and GC (Table 1). Following plasma protein preparation (see Supplementary Material), a total of 691 proteins were identified and quantified.

**Figure 2:**
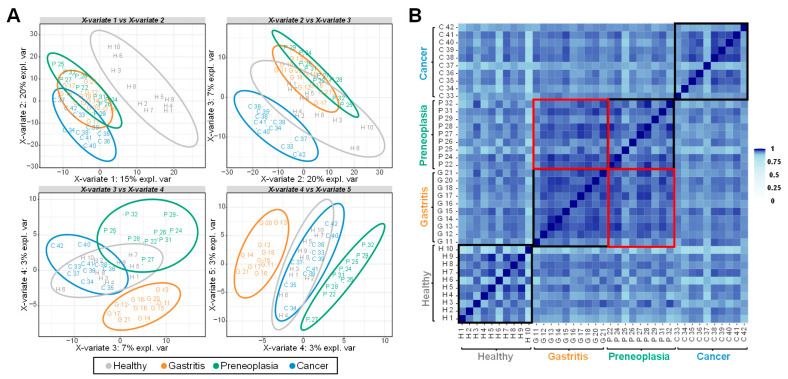
Multivariate analysis of the mass spectrometry-based proteomics data. (**A**) Sample plot of the PLS-DA model using five components. These components explain 48% of the total inertia. (**B**) Pairwise correlations between samples. Shades of blue represent correlation values. Black squares are sub-matrices corresponding to each category of patients. Red squares are the correlation matrices between samples of patients affected by gastritis and preneoplasia (AG/*p*).

To assess the similarity of proteomic profiles across groups, correlation analysis was performed (Figure 2). Overall, the proteomes showed high levels of correlation, with a minimum of 80.05%. Within-group correlation was particularly strong, especially among patients in the same diagnostic category. Notably, proteomes from gastritis and preneoplasia patients were closely aligned, whereas those from healthy individuals and GC patients were more distinct from other groups of pathology. Partial Least Square-Discriminated Analysis (PLS-DA) was used to further explore group separation based on proteomic profiles. The first two PLS-DA components clearly distinguished healthy individuals from patients affected by a pathology. Components 2 and 3 allowed for the distinguishing of GC patients from other patients (Figure 2A). As observed from the correlation analysis (Figure 2B), the separation between patients with gastritis and those with preneoplasia is low but still occurs at the level of the fourth component of the PLS-DA that explains only 3% of the total variance (Figure 2A). Together, the first four PLS-DA components allowed for the discrimination of the different patient categories based on their proteomes. These findings indicate distinct proteomic signatures in plasma samples associated with each group of patients.

### 2.3. Biomarker Candidates Identified by LC-MS/MS-Based Data

To identify candidate biomarkers capable of distinguishing between patient groups, we performed differential analyses using multiple moderated *t*-tests for all pairwise comparisons (“*Two by two*” comparisons), as well as a sparse PLS-DA analysis, as described in Section 4.

**Figure 3:**
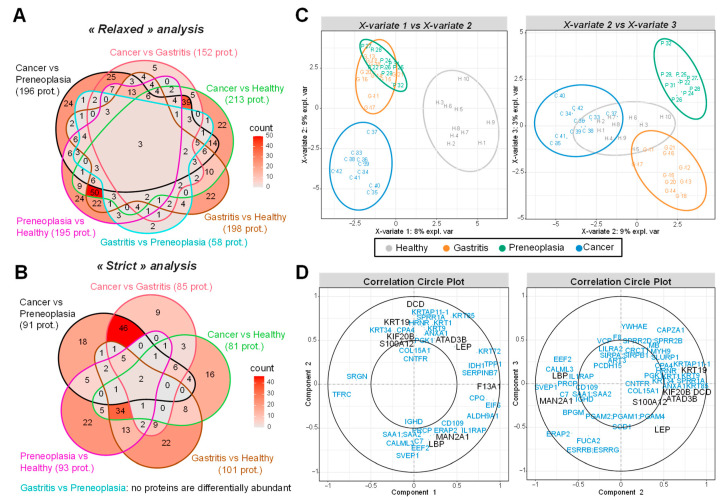
Identification of protein biomarkers from mass spectrometry-based proteomics data. (**A**) Venn diagrams summarizing the 408 biomarkers found in all the comparisons when imposing at least two quantified values in a category of patients («relaxed» analysis): 309 are differential in at least two comparisons. (**B**) Venn diagrams summarizing the 236 biomarkers found in all the pairwise comparisons when imposing at least nine quantified values in a category of patients («strict» analysis): 149 are in at least two comparisons. (**C**) Sample plot of the sparse PLS-DA model with three components. These components explain 20% of the total inertia. Each plot represents the samples projected on two axes, each one corresponding to a latent component of the model. (**D**) Correlation circle plot of the sparse PLS-DA. The proteins measured by ELISA test are highlighted in black.

*“Two by two” comparisons.* For comparison of one pathology vs. another, two types of statistical analyses were conducted: a “relaxed” analysis by imposing for each protein, at least two quantified values in the plasma samples of patients from one of the two compared categories (Figure 3A), and a “strict” analysis by imposing at least nine quantified values in one of the two compared categories (Figure 3B). Since these proteins are quantified in almost all patients in a category, we can expect the “strict” analysis to give more robust results. Six different comparisons were considered: each group of patients vs. healthy, gastritis vs. preneoplasia, and gastritis or preneoplasia vs. GC. From the pairwise comparisons resulting from the “relaxed” analysis (Figure 3A), 408 proteins were found significantly more abundant in one category of patients than in another, among which there were 309 with differential level in at least two comparisons (see Supplementary Figure S2 and Additional File S1). With the “strict” analysis approach (Figure 3B), 236 proteins show differential level comparing two groups: 149 are differential in at least two comparisons (see Supplementary Figure S3 and Additional File S2). It should be noted that the comparison of preneoplasia and gastritis categories shows the fewest proteins differentially abundant, with 58 differential proteins found in the “relaxed” analysis (Figure 3A, Supplementary Figure S2 and Additional File S1), while none are found with the “strict” analysis (Figure 3B, Supplementary Figure S3 and Additional File S2). Considering the “strict” analysis, two proteins appear with differential level in five comparisons over six: Transferrin Receptor Protein 1 (TFRC) and Immunoglobulin heavy constant g1 (IGHG1); eight proteins in four comparisons over six: Lysosomal Pro-X carboxypeptidase (PRCP), Serum amyloid A-1 protein (SAA1), Serum amyloid A-2 protein (SAA2), Serine protease 3 (PRSS3), a-mannosidase 2A1 (MAN2A1), Dermcidin (DCD), *Glycosylphosphatidylinositol phospholipase D* (GPLD1) and Interleukin-1 receptor accessory protein (IL1RAP). These proteins could serve as promising biomarker candidates for distinguishing between patient categories. In addition to pairwise condition comparisons, we performed a sparse PLS-DA analysis to identify a refined set of proteins capable of distinguishing all patient groups effectively.

*Sparse PLS-DA*. Unlike the previous approach that compares each category of patients two by two, this sparse PLS-DA analysis attempts to separate all the groups according to a restricted number of proteins: we imposed the choice of only ten biomarkers by component of the PLS-DA. The first component distinguishes healthy subjects from others, while the second one separates GC patients from those with gastritis and preneoplasia (Figure 3C). The third component allows a separation between gastritis and preneoplasia. The correlation circle plots highlight the correlation of each protein selected by the sparse PLS-DA with its different components (Figure 3D). Among the 85 proteins identified as potential biomarkers by the sparse PLS-DA analysis, Coagulation factor XIII A chain (F13A1) is highly correlated with the first component, while TFRC is inversely correlated. They could thus be biomarkers to separate healthy individuals from others. Other proteins like DCD, Keratin type I cytoskeletal 19 (KRT19), Kinesin-like protein KIF20B (KIF20B), ATPase family AAA domain containing 3B (ATAD3B), S100 calcium-binding protein A12 (S100A12), or Leptin (LEP) are positively correlated with the second component while MAN2A1, Lipopolysaccharide-binding protein (LBP), or Sushi Von Willebrand Factor Type A, EGF, and Pentraxin Domain containing 1 (SVEP1) are inversely correlated. Thus, these proteins could contribute to separate GC patients from gastritis and preneoplasia patients. When examining proteins correlated with the third component separating gastritis and preneoplasia, Capping Actin Protein of Muscle Z-Line Subunit a (CAPZA1), a-L-Fucosidase 2 (FUCA2), Endoplasmic Reticulum Aminopeptidase 2 (ERAP2), and LEP are found. They are also positively or negatively correlated with the second component separating GC patients from others. Interestingly, LEP is the only protein kept by the sparse PLS-DA with an absolute correlation close or superior to 0.5 with the three components. It was also found differentially abundant in the “relaxed” analysis in four comparisons (GC vs. gastritis, GC vs. healthy, preneoplasia vs. healthy, and GC vs. preneoplasia) (Supplementary Material Additional File S1) and could thus be an interesting candidate. Importantly, most of the proteins highlighted by the sparse PLS-DA are categorized as potential prognostic biomarkers of diverse cancers in the Human Protein Atlas (https://www.proteinatlas.org (accessed on 26 March 2025)) [23], thus confirming their relevance in our study and their potency as biomarker candidates associated with gastric preneoplasia or GC.

*Short-listing of 15 biomarker candidates from the LC-MS/MS analysis*. Based on the analysis of LC-MS/MS data, 15 proteins were short-listed for further confirmation by ELISA, considering both the results of the statistical analyses, their biological functions, and their relevance in cancer (Table 2). Statistically, all these proteins were found differentially abundant either in the “GC vs. preneoplasia” or “GC vs. heathy” comparison using the “strict” statistical analysis and could thus be biomarker candidates of GC. They were also significantly differentially abundant in at least one other comparison when using the “strict” statistical analysis, except LEP which presents some missing values that exclude it from this analysis. However, LEP was also particularly interesting to test by ELISA, according to the sparse PLS-DA results (Figure 3D). In addition, 7 of these 15 proteins were also differentially abundant between patients affected by preneoplasia and healthy controls in the “relaxed” statistical analysis (Carbonic anhydrase 2 (CA2), DCD, F13A1, Haptoglobin (HPT), Insulin-like growth factor-binding protein complex acid labile subunit (IGFALS), KRT19, and MAN2A1) and could thus be potential biomarkers of gastric preneoplasia.

### 2.4. ELISA-Based Validation of 15 Biomarker Candidates Highlighted by LC-MS/MS

ELISAs were used to measure the plasma concentrations of selected biomarker candidates in all samples of the cohort (n = 138) (Figure 1).

**Figure 4:**
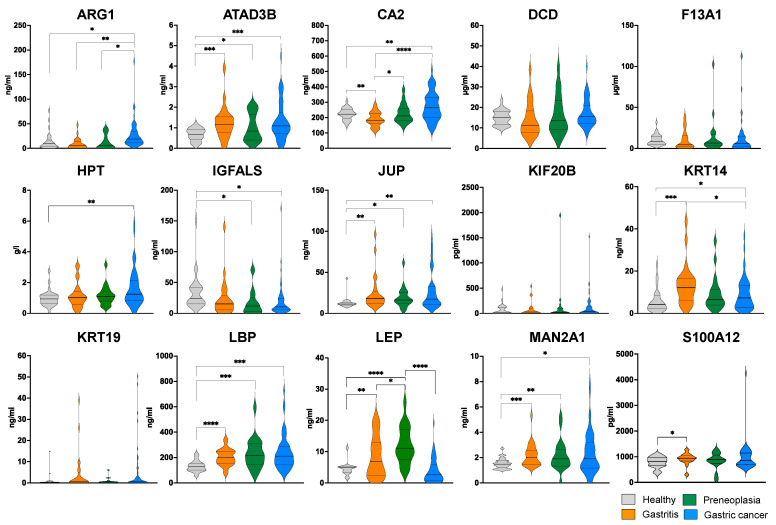
ELISA quantified plasma levels of 15 protein candidates from MS analysis to distinguish patient groups. Protein levels were measured by ELISA commercial assays as indicated in the methods section, on all samples of the cohort. Statistical analysis was performed using Mann–Whitney test. Only significant differential levels are indicated (**** *p* < 0.0001; *** *p* < 0.001; ** *p* < 0.01; * *p* < 0.05).

ELISA results confirmed LEP as a potent biomarker, in agreement with the findings from LC-MS/MS analyses. LEP plasma level is differentially abundant between patients with gastritis or preneoplasia vs. healthy individuals, and patients with preneoplasia vs. those with gastritis or GC (Figure 4). It is higher in the group of patients with preneoplasia than in the other categories and significantly lower in men than women for all groups, except GC (Figure S4). These findings suggest that plasma LEP concentration could serve as a robust biomarker for identifying preneoplastic lesions, in agreement with previous studies [24,25].

Differential plasma levels between patients with preneoplasia and healthy subjects were also observed for ATAD3B, IGFALS, Junction Plakoglobin (JUP), LBP, and MAN2A1 (Figure 4), which, along with Arginase-1 (ARG1), CA2, HPT, and KRT14, are also differentially abundant between GC patients and healthy individuals. Gender affects the plasma concentration of differentially abundant ATAD3B, JUP, and MAN2A1, specifically in women between each group of patients and healthy individuals (Figure S4). When comparing GC patients to healthy individuals, differential plasma levels are observed in women for HPT and in men for IGFALS. Age can also have an impact as in the case of ARG1, which is more abundant in older GC patients than in others (Figure S5). These nine proteins with diagnostic properties modulated by age or gender could thus be considered as potent biomarkers of gastric preneoplasia or GC.

We also assessed the heterogeneity within the preneoplasia group, which included patients with AG, IM, and DYS, by comparing biomarker concentration levels across these subgroups. No statistically significant differences were observed between them, reinforcing that this group represents closely related gastric lesions (Figure S6). However, the small sample size within each of these subgroups limits the statistical power to detect potentially meaningful differences in biomarker levels between these distinct pathological stages.

Although some of these proteins showed a promising trend, defining specific concentration thresholds for reliably diagnosing a given pathology in individual patients remains challenging. As reported in previous studies, single protein biomarkers often lack sufficient discriminatory power on their own. However, their diagnostic accuracy can be significantly enhanced when combined with other candidate biomarkers [22]. Next, we investigated the relationships between biomarkers concentration and evaluated the added value of multi-marker combinations to improve patient classification.

### 2.5. Correlations Between Plasma Concentration Levels of the Biomarkers

**Figure 5:**
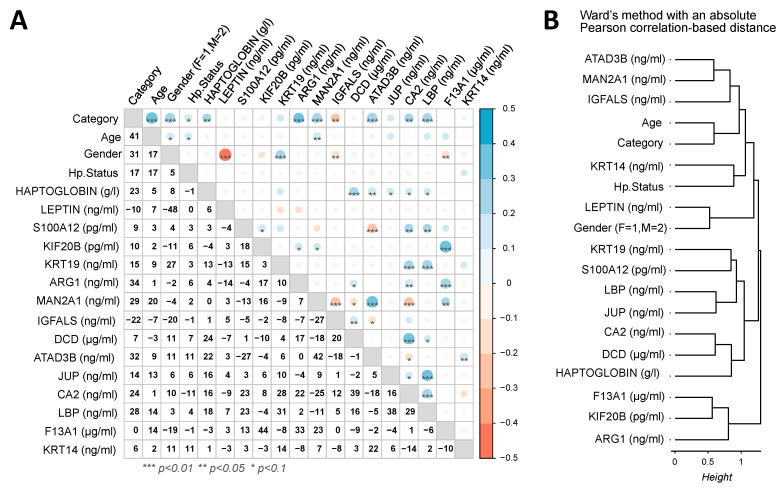
Correlations between biomarker concentrations measured by ELISA across patient samples. (**A**) Pearson correlation matrix between plasma concentration levels of the biomarkers. (**B**) Hierarchical clustering of biomarkers using a correlation-based distance and the Ward method.

Pearson correlation coefficients analysis between the biomarker concentrations in patients showed that many of them appear strongly significantly different from zero with a *p*-value threshold of 1% (Figure 5A). Many of the biomarkers are positively correlated. Their plasma concentration evolves globally similarly, as KRT19, CA2, and LBP between them, HPT with DCD, DCD with CA2, JUP with LBP, KIF20B and ARG1 with F13A1, and MAN2A1 with ATAD3B. Inversely, MAN2A1 is negatively correlated with IGFALS and CA2, as S100A12 and ATAD3B, or LEP with patient gender (i.e., higher levels in women than men). These observations led to four main protein clusters when using hierarchical clustering based on an absolute Pearson correlation distance (Figure 5B). The first cluster contains KRT19, S100A12, LBP, JUP, CA2, DCD and HPT, the second LEP and gender; the third includes age and *H. pylori* infection status of the patient with ATAD3B, MAN2A1, IGFALS and KRT14, while the fourth is composed of F13A1, KIF20B and ARG1. These different behaviors of biomarkers therefore indicate that not all biomarkers provide the same information. The use of their combination can thus be a positive strategy leading to a more accurate classification of patients.

### 2.6. Performance of Prediction Models with Single and Multiple Biomarkers

**Figure 6:**
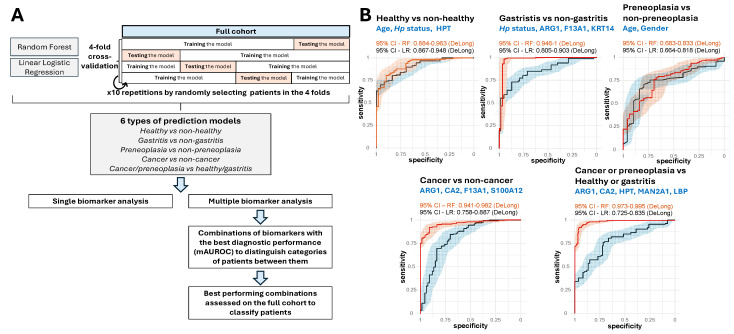
Prediction models with single and multiple biomarkers. (**A**) Schematic representation of the 4-fold cross-validation strategy repeated ten times, used to evaluate prediction models and methods used to identify the combinations of biomarkers with the best mAUROC values. (**B**) ROC curves assessed from the full cohort based on models using biomarkers with the best mean AUROC obtained from repeated cross-validations. 95% CI = 95% confidence interval. RF = random forests (orange), LR = linear logistic regression (black). AUROC values and ROC curves have been obtained using the pROC R package version 1.18.5. Confidence intervals have been obtained with the DeLong method. *Hp: H. pylori*.

As described in the Materials and Methods section, six types of prediction models were developed using either linear logistic regression or random forests, evaluated through 4-fold cross-validation repeated ten times (Figure 6A). Models were evaluated based on the AUROC criterion, as calculated from the test sets of the cross-validation process (Supplementary Material Additional File S3). We first evaluated each of the 15 ELISA-validated protein biomarkers individually, including age, gender, and *H. pylori* infection status as additional factors thus resulting in 18 biomarkers that can be used in our prediction models to eventually improve their performance.

*Single biomarker analysis.* We first assessed the predictive performance of individual protein biomarkers incorporated into the models. Table 3 reports the mean AUROC (mAUROC) values for all biomarkers across different models, where the reported mAUROC corresponds to the highest value obtained between logistic regression and random forest models (see also Supplementary Material Additional File S3). For models predicting all patient categories, the best-performing biomarker was the plasma concentration of KRT14 (mAUROC = 63.3%, 54.7–71.9% (95% CI)), closely followed by age (mAUROC = 63.2%, 53.8–72.6% (95% CI)). Notably, age emerged as a crucial factor, yielding the highest mAUROC for several classification tasks, including healthy vs. non-healthy (mAUROC = 80.1%, 66.6–93.6% (95% CI)), preneoplasia vs. non-preneoplasia (mAUROC = 71.8%, 54.6–89.0% (95% CI)), and cancer or preneoplasia vs. healthy or gastritis (mAUROC = 75.1%, 59.2–91.0% (95% CI)). These results reflect the structure of our cohort with healthy individuals younger than patients (Table 1). For cancer vs. non-cancer prediction, the best-performing biomarker was the plasma concentration of CA2 (mAUROC = 78.1%, 61–95.1% (95% CI)). Meanwhile, for gastritis vs. non-gastritis classification, the highest mAUROC was again obtained with KRT14 (mAUROC = 72.3%, 55.8–88.8% (95% CI)). Although these results demonstrate the potent utility of single biomarkers, their moderate mAUROC values indicate that individual biomarkers alone may not be sufficient for robust disease classification.

*Multiple biomarker analysis.* To improve predictive accuracy, we examined models incorporating multiple biomarkers using the same methodology (Figure 6A). Given that the number of possible biomarker combinations increases exponentially with the number of biomarkers included (Table 4), we limited our analysis to a maximum of six biomarkers selected from the 18 candidates, resulting in a total of 31,179 unique tested combinations (Supplementary Material Additional File S3). As reported in Table 4, models that integrated multiple biomarkers outperformed those using a single one, achieving higher mAUROC values. However, adding more biomarkers did not always lead to better performance, as none of the highest mAUROC values were observed in models containing six biomarkers. For models predicting all patient categories, the best-performing model achieved an mAUROC of 77.9% (62.8–93.0% (95% CI)) and incorporated age, gender, *H. pylori* status and the plasma concentration of ARG1 and KRT14. A different model, designed to predict cancer or preneoplasia vs. healthy or gastritis, yielded an even higher mAUROC of 83.9% (73.9–93.9% (95% CI)) and was based on the plasma concentrations of ARG1, HPT, CA2, MAN2A1, and LBP. HPT also emerged as a key biomarker to distinguish between healthy and pathological states, as combining with age and *H. pylori* status, this model achieved the highest mAUROC of 88.0% (77.6–98.3% (95% CI)). For cancer vs. non-cancer prediction, the top-performing model included the plasma concentrations of ARG1, CA2, F13A1, and S100A12, achieving an mAUROC of 85.3% (72.8–97.8% (95% CI)). The best model for gastritis vs. non-gastritis classification combined *H. pylori* status with the plasma concentrations of ARG1, KRT14, and F13A1, yielding an mAUROC of 82.2% (64.1–99.9% (95% CI)). For preneoplasia vs. non-preneoplasia prediction, the strongest model was based on the patient age and gender (mAUROC = 75.7%, 58.2–93.1% (95% CI)), closely followed by a model that also incorporated the plasma concentration of KRT14 (mAUROC = 74.4%, 53.6–95.2% (95% CI)). Thus, predicting preneoplasia vs. other categories appears more challenging even if the highest mAUROC values exceed 50%, indicating interesting predictive capability.

Our analysis revealed that age, gender, and *H. pylori* status significantly influenced the levels of several proteins (Figure 5, Figures S3 and S4). As discussed in Section 2.4, LEP showed gender differences, with higher levels in women than in men, while ATAD3B, JUP, and MAN2A1 showed differential abundance profiles by gender. Age effects were particularly notable for ARG1, with higher levels in older GC patients. Rather than simply adjusting for these potential confounders, we systematically incorporated them as additional predictors in our models. For each prediction task, we tested the impact of adding these demographic variables on model performance (Supplementary Materials, Additional File S3 and Table S2). This approach revealed task-specific trends: while demographic variables significantly improved the performance in distinguishing healthy from unhealthy individuals (age and *H. pylori* status with HPT: mAUROC = 88.0%), protein panels for cancer detection (ARG1, CA2, F13A1, S100A12: mAUROC = 85.3%) and cancer/preneoplasia discrimination (ARG1, CA2, HPT, MAN2A1, LBP: mAUROC = 83.9%) achieved superior performance without demographic variables, indicating that these biomarkers capture disease-specific signals beyond the effects of age and gender. For multi-category classification, combining demographic factors with protein markers (age, gender, *H. pylori* status, ARG1, KRT14: mAUROC = 77.9%) provided optimal performance. This systematic integration of confounding variables made it possible to select the most robust biomarker combinations while taking demographic influences into account.

*Full cohort validation.* Finally, we assessed the best-performing models on the full cohort to obtain final performance estimates. Using the combinations of biomarkers displaying the best mAUROC values with repeated cross-validations, we assessed their performance to classify patients on the full cohort using linear logistic regression and random forests (Figure 6A). Notably, the AUROC values for the models classifying cancer vs. non-cancer, and cancer/preneoplasia vs. healthy/gastritis, were estimated superior to 94.1% and 97.3%, respectively, using random forests (lower bounds of 95% CI) (Figure 6B). This demonstrates that the combinations of short-listed biomarkers corresponding to ARG1, CA2, F13A1, and S100A12 for the distinction of cancer vs. non-cancer (AUROC 94.1–96.2% (95% CI)) and ARG1, CA2, HPT, MAN2A1, and LBP to distinguish cancer or preneoplasia vs. healthy or gastritis (AUROC 97.3–99.5% (95% CI)), enable accurate classification of patients for these tasks.

## 3. Discussion

GC is a multifactorial disease often diagnosed at an advanced stage, limiting the efficacy of therapeutic interventions and contributing to its poor prognosis. Currently, diagnosis relies on upper endoscopy, an invasive and resource-intensive procedure that requires specialized equipment and highly skilled clinicians to accurately identify gastric preneoplasia, particularly DYS which need a periodical surveillance [26,27]. A critical gap is the lack of non-invasive diagnostic methods for early detection of GC. In this context, blood-based biomarkers represent a promising alternative to overcome the limitations of endoscopic screening and to reduce the global burden of GC.

In this study, we used bottom-up LC-MS/MS-based proteomics of plasma samples from patients with gastric lesions at various stages of the carcinogenesis cascade, followed by ELISA validation, to identify and confirm plasma protein biomarkers associated with gastric preneoplastic and cancerous lesions. Our results provide compelling evidence that specific protein concentration profiles in plasma can serve as reliable indicators of disease stage and may be combined to develop non-invasive diagnostic models capable of identifying individuals at high risk of GC, even at asymptomatic stages.

Our LC-MS/MS analyses highlighted numerous candidate biomarkers across patient categories (408 differentially abundant proteins in the “relaxed” statistical analysis and 236 in the “strict” analysis). These proteins are involved in pathways known to be dysregulated in cancer, as annotated in KEGG (https://www.genome.jp/kegg/pathway.html (accessed on 26 March 2025)) and the Human Protein Atlas (https://www.proteinatlas.org (accessed on 26 March 2025)) databases, including proteins related to signaling and cellular processes (DCD, IL1RAP, KRT14, KRT19, S100A12, SAA1/SAA2, SVEP1, TFRC), innate immunity and immune response (F13A1, IGHG1, LBP), cell motility and membrane trafficking (CAPZA1, KIF20B), glycan biosynthesis (FUCA2, MAN2A1), digestive and cancer-related systems (CA2, JUP, LEP, PRCP, PRSS3), metabolism, cell energy and growth (ARG1, ATAD3B, ERAP2, GLPD1, HPT, IGFALS). Most of these proteins are recognized as potential prognostic biomarkers in liver (TFRC and GPLD1), renal (IGHG1, PRCP, SAA1/2, and IL1RAP) and digestive (PRSS3) cancers in the Human Protein Atlas database. Importantly, the GC proteomics database (GCPDB) [28] has previously reported differential levels of IGFALS, LBP, S100A12, F13A1, SAA1/SAA2, PRCP, and IGHG1 in plasma/serum proteome MS analysis of GC patients at various stages, further validating the relevance of our findings.

From our discovery set, fifteen proteins were short-listed, among which several emerged as promising biomarkers after validation by ELISA in the entire cohort of 138 subjects (including 106 patients). Of note, LEP displayed significant differential abundance across patient groups, with the highest levels in patients with preneoplasia. Given its diverse functions in the digestive system, including the regulation of immune response, cell proliferation and tissue repair, and lipid and glucose metabolism [29], LEP represents a biomarker of special interest for gastric preneoplastic processes. Previous studies have already associated higher LEP levels with gastric inflammation and IM [24], supporting our findings. Additional proteins, ATAD3B, IGFALS, JUP, LBP, and MAN2A1, also displayed significantly higher plasma concentration in the preneoplasia group compared to healthy individuals, suggesting them as early markers of gastric mucosal transformation.

When comparing GC patients to healthy individuals, nine proteins (ARG1, ATAD3B, CA2, HPT, IGFALS, JUP, KRT14, LBP, and MAN2A1) displayed significantly altered plasma levels. These proteins have established roles in carcinogenesis. ARG1 modulates oncogenic signaling pathways such as PI3K/AKT/mTOR, STAT3, and MAPK [30]. It has also been mentioned to be enriched in hepatocellular carcinoma [31]. ATAD3B, a mitochondrial membrane-bound protein, has been associated with tumorigenesis [32] and suggested as a potential prognostic biomarker for hepatocellular carcinoma patients [33]. The low expression of CA2, a metalloenzyme involved in gastric acid secretion [34], correlates with poor prognosis in GC patients [35]. HPT, a hemoglobin-binding glycoprotein associated with inflammatory response, shows aberrant glycosylation in GC, suggesting it as a signature molecule for GC diagnosis and detection [36]. It has also been previously proposed as a biomarker to predict colorectal cancer and tumor progression [37]. IGFALS, crucial for the stability of circulating insulin growth factors, is down-regulated in hepatocellular carcinoma and associated with poor prognosis [38]. JUP, a cell–cell junction protein and transcription factor, exhibits loss correlated with GC malignancy and poor prognostics [39]. KRT14, a member of the keratin family of intermediate filament proteins, could be an indicator of the remodeling of the gastric epithelium, promoting tumor growth and invasion [40]. LBP has been linked to GC pathogenesis and proposed as both a prognostic [41] and a diagnostic biomarker for liver metastasis in GC [42]. MAN2A1, involved in N-linked oligosaccharide processing in the Golgi apparatus, may contribute to the metabolic reprogramming and altered glycosylation characteristic of cancer cells. It has been proposed as a potential biomarker significantly related to prognosis and lymph node metastasis in colorectal cancer patients [43].

While individual biomarkers showed encouraging trends, the heterogeneity in expression patterns and inter-patient variability prevented accurate classification based on single protein measurements. Our correlation analysis revealed distinct biomarker clusters, suggesting that combining multiple markers could enhance classification accuracy and disease stage differentiation. From the prediction models-based analysis, two panels proved particularly effective: ARG1, CA2, HPT, MAN2A1, and LBP (mAUROC: 83.9%; 73.9–93.9% (95% CI)) for differentiating patients with cancer or precancerous lesions from healthy individuals or patients with gastritis; and ARG1, CA2, F13A1 and S100A12 (mAUROC: 85.3%; 72.8–97.8% (95% CI)) for differentiating GC from non-cancer patients. ARG1 and CA2 appeared in both panels, reinforcing their significance as GC biomarkers. Interestingly, while S100A12 and F13A1 did not show significant individual differences between GC patients and healthy individuals, they enhanced diagnostic performance when combined with ARG1 and CA2. S100A12, a member of the calgranulin protein family, is related to innate immunity and has been proposed as a prognostic factor in GC [44], while F13A1 has been associated with tumor metastasis in lung cancer [45] and poor prognosis in several cancers [46,47,48].

According to their biological function, these seven selected biomarkers form a functionally interconnected network representing three interrelated pathological processes: inflammatory signaling, metabolic reprogramming, and immune modulation. The inflammatory component includes S100A12, LBP, and HPT. S100A12 functions as a damage-associated molecular pattern that activates pro-inflammatory signaling through RAGE and TLR4 receptors [49]. LBP, an acute-phase protein, facilitates pathogen recognition and inflammatory responses by activating TLR4 receptors [50]. HPT is another acute-phase protein that modulates immune responses and serves as a link between inflammation and cancer progression [36]. The metabolic adaptation component is represented by CA2 and MAN2A1. CA2, identified as a biomarker in gastrointestinal tumors [51], plays a critical role in regulating intracellular pH homeostasis despite the acidic extracellular tumor environment. This pH regulation is essential for cancer cell survival, proliferation, proper protein folding, and glycosylation. MAN2A1 is involved in N-glycan maturation. Its inhibition has been reported to enhance cancer cell sensitivity to T-cell-mediated killing through alterations in the glycosylation patterns of immune checkpoint proteins [52]. The immune modulation component includes ARG1 and F13A1. ARG1 functions as a metabolic checkpoint in immune cells by depleting arginine, creating an immunosuppressive environment that facilitates tumor immune evasion [53]. F13A1 contributes to tissue remodeling processes that can be co-opted during tumor invasion and metastasis, potentially modifying the tumor microenvironment [54]. These pathological processes may be integrated through multiple mechanistic interconnections. For instance, a functional relationship between LEP, ARG1, and MAN2A1 can be postulated. While LEP is not among the seven biomarkers of the signatures, it appears overexpressed in preneoplasia patients. LEP has been shown to function as a systemic nutritional checkpoint that enhances macrophage inflammatory responses through mTORC2-dependent signaling [55]. In the gastric microenvironment, LEP may thus upregulate ARG1 expression via the PI3K/Akt/mTOR pathway, creating an immunosuppressive niche. Separately, MAN2A1 plays a critical role in N-glycan maturation, which has implications for cellular signaling pathways. N-linked glycans in specific domains of the LEP receptor are critical for optimal LEP binding and receptor function [56]. We can thus hypothesize that these proteins may be part of interconnected pathways during gastric carcinogenesis process.

While our identified biomarkers show promise for GC detection, it is important to acknowledge that many are not exclusive to gastric pathology. However, the strength of our approach lies in their specific combination rather than individual proteins. This suggests that coordinated alterations create distinctive signatures that may be more specific to gastric pathology. Future studies comparing these signatures across different gastrointestinal and inflammatory conditions would further clarify their diagnostic specificity.

Despite the identification of promising biomarker panels achieving high AUROC values, limitations should be acknowledged. First, our single-country cohort cannot integrate the genetic and environmental diversity specific to each population and known to modulate the GC incidence worldwide. Second, variations in age, gender distribution, and *H. pylori* status across groups of the cohort are potential confounders. Although this analysis demonstrated that our protein panels capture disease-specific signals independent of these factors, these imbalances remain as methodological limitations. Third, our clinic-based recruitment, rather than population-based sampling, may introduce selection bias, potentially overestimating the diagnostic performance that would be observed in a general screening population. Fourth, the preneoplasia group’s heterogeneity (combining AG, IM, and DYS patients) represents a methodological limitation. Although we found no significant differences in biomarker levels between these conditions, the limited sample sizes in each subgroup warrant caution in interpretation. This heterogeneity likely contributes to the lower discriminatory performance for preneoplasia classification. While the proteins in our panels have previously been linked to GC in independent studies, supporting our findings, formal validation in independent cohorts are still necessary before considering clinical implementation. Future studies must consolidate these findings in diverse populations with longitudinal follow-up to evaluate the potential of these biomarker panels in real-world screening settings.

Clinical implementation of our blood-based biomarker panel could follow a hierarchical approach: (1) Initial screening using our model for distinguishing healthy from non-healthy individuals (combining age, *H. pylori* status, and HPT; mAUROC 88.0%); (2) For individuals classified as non-healthy, application of a second model to distinguish between low-risk (gastritis) and high-risk (preneoplasia/cancer) conditions (using ARG1, CA2, HPT, MAN2A1, and LBP; mAUROC 83.9%); (3) Finally, for those classified as high-risk, application of a third model to distinguish cancer specifically (using ARG1, CA2, F13A1, and S100A12; mAUROC 85.3%), with endoscopic evaluation recommended only for those with positive results. This approach would be proposed as a first intent for risk stratification, rather than as a replacement for endoscopy itself. Only in the case of a positive evaluation for high risk of preneoplasia and/or GC, endoscopy will be proposed to patients. Screening intervals of 3–5 years for general populations and more frequent testing (1–2 years) for those with risk factors or previously detected for gastric preneoplasia would be in alignment with endoscopic surveillance guidelines [57]. By reducing unnecessary procedures, it could improve the cost-effectiveness of GC screening while maintaining appropriate surveillance for high-risk individuals. Implementation would be straightforward through existing blood sampling infrastructure, providing the advantages of non-invasive testing and enabling wider population coverage as well as the earliest detection of patients leading to reduce the global burden of this aggressive cancer.

From a public health perspective, our findings have far-reaching implications. Implementation of blood-based testing could improve screening participation in both high-risk populations and resource-limited settings where endoscopic capacity is insufficient. In countries with established screening programs (i.e., Japan, South Korea), our approach could help refine risk stratification, while in Western countries with no screening strategies for GC, this test could enable more targeted surveillance, particularly for individuals not identified as high-risk who are often detected at late stages. To conclude, our biomarker panels represent a significant step toward accessible, cost-effective tools that could transform GC screening paradigms globally.

## 4. Materials and Methods

### 4.1. Study Population

The study cohort consists of 138 participants. It includes 32 healthy asymptomatic volunteers more than 18 years old, recruited at the Institut Pasteur clinical investigation and biomedical research support unit (ICAReB-Clin). All were confirmed for their *H. pylori*-negative serology using a commercial ELISA (Serion/diagnostic, Wurzburg, Germany). The groups of patients included 26 cases of non-AG, referred to as “gastritis”, and 20 of AG/preneoplasia, referred to as the “preneoplasia” group, including patients with AG either with IM or DYS or not (Table 1). Patients with gastritis and preneoplasia were recruited at the unit of Hepato-Gastroenterology, AP-HP, A. Paré Hospital, Boulogne-Billancourt, France. The 60 patients with GC (stages III and IV) were enrolled from the unit of Digestive Oncology, AP-HP, European Hospital G. Pompidou (HEGP), Paris, France. Non-inclusion criteria for healthy volunteers and patient recruitment included individuals with familial history of GC, or suffering from chronic inflammatory disease or autoimmune disorders, or previously treated by antibiotherapy or anti-inflammatory drugs for at least two weeks before inclusion. Any suspicion of stomach dysfunction is a non-inclusion criterion for healthy controls. All patients were previously diagnosed through upper endoscopy combined with gastric biopsies and anatomopathology analysis. All patients were adults (>18 years old), they had not received antibiotherapy and had not been treated with bismuth compounds, proton pump inhibitors, or non-steroidal anti-inflammatory drugs for at least two weeks prior to the study. Each patient was informed about the study and provided written consent. For each patient, 10 mL of blood was collected and transferred to Leucosep^®^ tubes (Dutscher, Bernolsheim, France) for plasma isolation according to the recommendation of the supplier.

The study was approved by a French competent Ethics Committee (CCPPRB IDF IV, Ref: 2014/19NICB, 27 March 2014), by the Institut Pasteur officials (Ref 2013-29; 7 February 2014), the French advisory committee on information processing in Health Research (Ref 14.202; 12 March 2014) and the French data protection authority: Commission Nationale de l’Informatique et des Libertés (CNIL) (Ref: 2017-204, 8 August 2017).

### 4.2. Identification and Quantification of Plasma Proteomes Using LC-MS/MS

*Plasma samples for LC-MS/MS analysis.* As indicated in Table 1, plasma proteome identification and quantification were performed on samples from a subset of cohort patients with gastritis (n = 10), preneoplasia (n = 9), GC (n = 10), and healthy controls (n = 10). The most abundant proteins were first depleted from plasma, and the remaining protein samples underwent proteolytic digestion, as detailed in the Supplementary Material. Next, protein identification and quantification were performed using Data-Independent Acquisition (DIA), based on a spectral library obtained through Data-Dependent Acquisition (DDA).

*LC-MS/MS analysis.* Mass spectrometry-based plasma proteomics were conducted in two steps. The first step was dedicated to the spectral library generation from DDA using the fractionated “pool” sample. The second step was defined to specifically identify and quantify plasma proteome for each patient group from DIA. For this purpose, a nanochromatographic system (Proxeon EASY-nLC 1200-Thermo Fisher Scientific, Waltham, MA, USA) was coupled on-line to a Q Exactive^TM^ HF Mass Spectrometer (Thermo Fisher Scientific) using an integrated column oven (PRSO-V1-Sonation GmbH, Biberach, Germany). For each sample, 1 μg of peptides was injected onto a home-made C18 column. Peptides elution and mass spectra acquisition were performed as described in the Supplementary Material.

Data processing for proteins identification and quantification including building of the spectral library and data analysis for DIA method are reported in the Supplementary Material.

### 4.3. Statistical Analysis of Large-Scale LC-MS/MS Proteomic Data for Biomarker Identification

*Multivariate analysis of the MS-based proteomics data.* Pairwise correlation analysis, hierarchical clustering and PLS-DA have been performed to highlight global similarities between quantified proteomes of plasma samples. The correlation matrix represents the Pearson correlation coefficients between each pair of samples computed using all complete pairs of intensity values measured in these samples. The hierarchical cluster analysis has been conducted via multiscale bootstrap resampling (1000 bootstrap replications) with Ward’s method and a correlation-based distance measure thanks to the *pvclust* function of the *pvclust* R package version 2.2-0 [58], after log2 transformation of the intensities, imputation of the missing values with the *impute.slsa* function of the *imp4p* R package version 1.2 [59] and a normalization using a sample-median centering method inside conditions. PLS-DA using five components was performed using the *mixOmics* R package version 6.26.0 [60] to assess whether the quantified proteomes differ among the four patient groups (Healthy, Gastritis, Preneoplasia, and GC).

*Identification of potential biomarkers from MS-based proteomics data*. To identify potential protein biomarkers, pairwise differential analyses (e.g., comparisons of one category of patient vs. another) were conducted, as well as a multivariate sparse PLS-DA.

To identify proteins that were more abundant in one condition than another, quantified intensities obtained using Spectronaut were compared. Proteins were retained for further statistical analysis only if they had at least two quantified intensity values (for the “relaxed” analysis) or nine quantified intensity values (for the “strict” analysis) in at least one of the two conditions being compared. Proteins that were quantified in one condition but not in the other were set aside, as they could be directly considered as potential biomarkers. Following this initial filtering, the intensities of the remaining proteins were log-transformed (log2). Next, intensity values were normalized by median centering within a same category of patients (Section 3.5 in [61]). Missing values were imputed using the *impute.slsa* function from the *imp4p* R package version 1.2 [59]. Proteins with a fold-change (FC) below 2 (i.e., absolute log_2_(FC) < 1) were considered not significantly differentially abundant. Statistical testing was then performed using a *limma t*-test, implemented in the *limma* R package version 3.58.1 [62]. To control for multiple testing, an adaptive-Hochberg procedure was applied to the resulting *p*-values using the *adjust.p* function from the *cp4p* R package version 0.3.6 [63]. The robust method described in [64] was used to estimate the proportion of true null hypotheses among the statistical tests. Proteins with an adjusted *p*-value below a false discovery rate (FDR) of 1% were considered significantly differentially abundant. Ultimately, proteins identified as potential biomarkers were those that emerged from this multiple testing analysis, along with those quantified in one condition but not in the other. The lists of potential biomarkers obtained from the different comparisons were then compared using Venn diagrams to highlight proteins that are differentially abundant in several comparisons.

A sparse PLS-DA was performed alongside pairwise comparisons of patient categories. Unlike the previous approach, which compares patient groups two by two, sparse PLS-DA aims to distinguish all groups simultaneously using a restricted set of proteins. For this analysis, only proteins with at least two quantified values in all patient categories were considered. Similarly to the differential analyses, the intensities of the selected proteins were log-transformed (log2), normalized by median centering within a same category of patients (Section 3.5 in [61]), and missing values were imputed using the *impute.slsa* function from the *imp4p* R package version 1.2 [59]. Finally, sparse PLS-DA was applied, with a constraint of selecting only ten proteins per component, using the *mixOmics* R package version 6.26.0 [60].

We employed multiple complementary analytical strategies to ensure robust biomarker identification. The «relaxed» analysis (requiring at least two quantified values in patient categories) maximized sensitivity for discovery of potential biomarkers, while the «strict» analysis (requiring at least nine quantified values) prioritized reproducibility and reliability. This dual approach allowed us to balance biomarker discovery with confidence in the findings. The sparse PLS-DA analysis provided a complementary multivariate perspective, identifying proteins capable of distinguishing all patient groups simultaneously rather than through pairwise comparisons. This multi-faceted statistical strategy strengthens confidence in our findings and provides more reliable performance estimates than single validations would allow.

### 4.4. Validation of LC-MS/MS-Identified Proteins Using Enzyme-Linked Immunosorbent Assays (ELISA), Statistical Analysis and Prediction Models

To validate the protein biomarker candidates deduced from the analysis of the LC-MS/MS data, the plasma concentration of 15 short-listed proteins was measured across the full cohort of patient plasma samples (138 samples) using ELISA according to the manufacturer’s instructions (MyBiosource, San Diego, CA, USA and R&D systems, Minneapolis, MN, USA) (Supplementary Material Table S1).

The final selection of 15 proteins for ELISA validation was based on (1) statistical significance in the LC-MS/MS analyses, particularly proteins showing differential abundance in multiple comparisons; and (2) biological relevance to cancer processes as documented in the literature and pathway databases. Statistical analysis of the concentration levels was performed using Student’s *t*-test or Mann–Whitney test. Results were considered significant if *p* < 0.05.

*Correlation analysis of measured concentrations.* The measured concentrations were analyzed using Pearson correlation coefficients between biomarkers, using all complete pairs of concentration values measured in these samples. A hierarchical cluster analysis has been conducted with Ward’s method and a correlation-based distance measure derived from the correlation coefficients. This approach was used to identify biomarkers displaying similar concentration patterns across patients.

*Estimation of prediction models to distinguish patient categories from measured concentrations.* To estimate prediction models for the different pathologies, we used a 4-fold cross-validation strategy repeated ten times. It consists of randomly splitting the set of patients into four sets while preserving the proportion of patients associated with the different pathologies in each as in the original dataset. Three sets of patients over four are then used alternatively to create a training dataset used to estimate the prediction model, while the fourth set is used to assess the performance of the estimated model (test dataset). For each tested combination of biomarkers, we included only patient samples with complete measurements for all biomarkers in the predictive modeling analyses. This complete case approach ensured the reliability of our multivariate models by eliminating potential bias from missing values. An up-sampling technique was used to duplicate patients of the minority categories with replacement to tackle the imbalance of the categories in the training dataset, in view of improving model performance. Finally, each model provides an estimated probability that a patient is affected by a pathology from a set of biomarkers. We assessed the results of the estimated prediction models on the remaining set of patients (test dataset) using AUROC. For each model, we randomly repeated, ten times, the 4-fold cross-validation strategy to assess the variations in results. This strategy not only assesses model performance but also mitigates the risk of overfitting. This cross-validation approach was applied to a focused set of proteins that were shortlisted based on our stringent FDR-controlled discovery analysis from MS-based proteomics data, allowing us to identify biomarker combinations with robust predictive power. AUROC values and ROC curves have been obtained using the pROC R package [65]. Confidence intervals have been obtained with the DeLong methods [66].

Different types of prediction models were estimated. First, we estimated a model predicting all categories of patients (i.e., predicting whether the patient is suffering from GC, preneoplasia, gastritis or if they are healthy). Second, we estimated four binary models specific to each category (i.e., predicting healthy vs. non-healthy, cancer vs. non-cancer, etc.). Third, we evaluated a model grouping GC and preneoplasia in one category and healthy and gastritis in another (i.e., predicting GC/preneoplasia vs. healthy/gastritis). This approach results in six types of prediction models; each of them is estimated by both linear logistic regression and using random forests, leading to twelve estimated models for each combination of biomarkers.

Our study design ensured adequate statistical power and model stability for the multivariate analyses presented. With sample sizes of 62 (Cancer or Preneoplasia) vs. 51 (Healthy or Gastritis) for our five-biomarker panel and 43 (Cancer) vs. 70 (non-Cancer) for our four-biomarker panel, we exceeded the conventional «rule of 10» that suggests a minimum of 10 observations per variable for reliable model estimation. Moreover, we employed repeated 4-fold cross-validation to safeguard against overfitting when estimating our multivariate models, further enhancing model stability. The post hoc power analysis has been conducted to detect large effect sizes (Cohen’s d = 0.8) with two sample *t*-tests in our key comparisons with the pwr.t2n.test R function of the pwr R package version 1.3-0 (https://CRAN.R-project.org/package=pwr (accessed on 6 October 2025)).

## 5. Conclusions

Our study presents a comprehensive approach for the early detection of GC and its precursor lesions by leveraging plasma proteomics. Based on predictive models achieving high classification performance using a minimal set of proteins, combinations of biomarkers with high diagnosis accuracy have been identified for distinguishing cancer from non-cancer (AUROC > 94%) and cancer or preneoplasia from healthy or non-atrophic gastritis cases (AUROC > 97%) within our study cohort. These findings pave the way to future development of a non-invasive and highly predictive blood test as a risk stratification tool to identify the earliest individuals at high risk of GC. While these results are highly promising, the next critical step is external validation in independent, multicenter cohorts to confirm its global use in clinic. If successfully validated, this diagnostic test could also be useful for the follow-up of patients previously detected with preneoplasia as well as to follow the recurrence or remission of patients under anticancer treatment, thus optimizing patient care and survival.

## Figures and Tables

**Table 1:** Characteristics of the study population. The gastritis group corresponds to patients with non-atrophic gastritis. The group of preneoplasia is composed of AG (21%), IM (47%), and IM and DYS (32%). GC group includes GC of intestinal type (53%), of diffuse type (37%) and undifferentiated (10%), among which stage II (2%), III (29%), and IV (69%). The exploratory LC-MS/MS analysis was performed on a subgroup of patients (numbers in italic). *p*-values for statistical analysis using Mann–Whitney test to compare age in healthy groups to other patient groups.

Group	n	Mean Age (Range)	Sex Ratio (M/F)	*H. pylori* Positive (%)
**Healthy**	32 *10*	42 (21–70) *38 (22–69)*	0.4 *0.4*	0 *0*
**Gastritis**	26	59 (27–88)	0.7	58%
		*p = 0.0003*		
			
	*10*	*38 (22–69)*	*0.6*	*58%*
		*p = 0.0015*		
**Preneoplasia**	20	70 (50–83)	0.4	61%
		*p < 0.0001*		
			
	*9*	*70 (54–83)*	*0.5*	*50%*
		*p = 0.0006*		
**Gastric cancer**	60	62 (29–84)	2	28%
		*p < 0.0001*		
			
	*10*	*62 (51–74)*	*4*	*40%*
		*p = 0.0044*		
**Total**	**138**			
	***39***			

**Table 2:** List of fifteen proteins from plasma proteomics analysis that were measured by ELISA. Name, uniprot code, description of proteins, KEGG pathways, and information from the Cancer resource of the Human Protein Atlas (HPA) are reported. The HPA Cancer resource provides information on mRNA expression, including whether a gene is enriched in a particular cancer type («Cancer enriched»: at least 4-fold higher mRNA level in a particular tissue/cell type compared to any other tissues/cell types; «Cancer enhanced»: at least 4-fold higher mRNA level in a particular tissue/cell type compared to the average level in all other tissues/cell types; or low cancer specificity), and whether it is («potential» or «validated») prognostic for patient survival in each cancer types from The Cancer Genome Atlas (TCGA). * High expression unfavorable; ** High expression favorable.

Protein Name	Uniprot Code	Protein Description	KEGG Pathways	Human Protein Atlas (TCGA)
**ARG-1**	P05089	Arginase-1	hsa01100 Metabolic pathways hsa00220 Arginine biosynthesis	*Cancer enriched* Liver Hepatocellular Carcinoma. No prognostic found.
**ATAD3B**	Q5T9A4	ATPase family AAA domain containing 3B	hsa03029 Mitochondrial Biogenesis	*Cancer enhanced* Testicular Germ Cell Tumor. *Potential prognostic* * Liver Hepatocellular Carcinoma, Kidney Renal Clear Cell Carcinoma.
**CA2**	P00918	Carbonic anhydrase 2	hsa00910 Nitrogen metabolism hsa01100 Metabolic pathways hsa04971 Gastric acid secretion	*Cancer enriched* Kidney Chromophobe Renal Cell Carcinoma. *Potential prognostic* * Lung Squamous Cell Carcinoma.
**DCD**	P81605	Dermcidin	hsa09193 Unclassified: signaling and cellular processes	*Cancer enriched* Breast Invasive Carcinoma. No prognostic found.
**F13A1**	P00488	Coagulation Factor XIII A Chain	hsa09151 Immune system hsa04610 Complement and coagulation cascades	*Low cancer specificity*. *Potential prognostic* * Lung Squamous Cell Carcinoma.
**HPT**	P00738	Haptoglobin	hsa09181 Protein families: metabolism hsa01002 Peptidases and inhibitors >Serine peptidases hsa04147 Exosomal proteins of other cancer cells	*Cancer enriched* Liver Hepatocellular Carcinoma. *Validated prognostic* * Kidney Renal Clear Cell Carcinoma, Stomach Adenocarcinoma Lung Adenocarcinoma. *Potential prognostic* ** Hepatocellular Carcinoma.
**IGFALS**	P35858	Insulin-like growth factor-binding protein complex acid labile subunit	hsa04935 Growth hormone synthesis, secretion and action hsa04147 Exosomal proteins of haemopoietic cells	*Cancer enhanced* Hepatocellular Carcinoma. *Potential prognostic* ** Hepatocellular Carcinoma, Lung Adenocarcinoma
**JUP**	P14923	Junction plakoglobin	hsa04820 Cytoskeleton in muscle cells hsa05200 Pathways in cancer hsa05202 Transcriptional misregulation in cancer hsa05221 Acute myeloid leukemia hsa05226 Gastric cancer hsa05412 Arrhythmogenic right ventricular cardiomyopathy	*Low cancer specificity*. *Potential prognostic* *, Hepatocellular Carcinoma *Potential prognostic* ** Kidney Renal Clear Cell Carcinoma.
**KIF20B**	Q96Q89	Kinesin-like protein KIF20B	hsa04814 Motor proteins hsa04131 Membrane trafficking hsa04812 Cytoskeleton proteins	*Low cancer specificity.* *Potential prognostic* * Lung Adenocarcinoma, *Potential prognostic* ** Kidney Renal Clear Cell Carcinoma *Validated prognostic* * Pancreatic Adenocarcinoma.
**KRT14**	P02533	Keratin, type I cytoskeletal 14	hsa04915 Estrogen signaling pathway hsa05150 *Staphylococcus aureus* infection hsa04147 Exosomal proteins of epithelial, colorectal cancer, melanoma cells	*Cancer enriched* Head and Neck Squamous Cell Carcinoma. *Potential prognostic* ** Breast Invasive Carcinoma *Potential prognostic* * Lung Adenocarcinoma.
**KRT19**	P08727	Keratin, type I cytoskeletal 19	hsa04915 Estrogen signaling pathway hsa05150 *Staphylococcus aureus* infection hsa04147 Exosomal proteins of epithelial, colorectal cancer, melanoma cells	*Low cancer specificity*. *Potential prognostic* * Pancreatic Adenocarcinoma. *Validated prognostic* * Kidney Renal Clear Cell Carcinoma.
**LBP**	P18428	Lipopolysaccharide- binding protein	hsa04064 NF-kB signaling pathway hsa04620 Toll-like receptor signaling pathway hsa04936 Alcoholic liver disease hsa05152 Tuberculosis hsa05417 Lipid and atherosclerosis hsa04147 Exosomal proteins of other cancer cells	*Cancer enriched* Liver Hepatocellular Carcinoma. *Validated prognostic* * Kidney Renal Clear Cell Carcinoma *Potential prognostic* * Kidney Renal Papillary Cell Carcinoma.
**LEP**	P41159	Leptin	hsa04060 Cytokine–cytokine receptor interaction hsa04080 Neuroactive ligand–receptor interaction hsa04081 Hormone signaling hsa04152 AMPK signaling pathway hsa04630 JAK-STAT signaling pathway hsa04920 Adipocytokine signaling pathway hsa04932 Non-alcoholic fatty liver disease	*Cancer enhanced* Breast Invasive Carcinoma. No prognostic found.
**MAN2A1**	Q16706	Alpha-mannosidase 2	hsa00510 N-Glycan biosynthesis hsa00513 Various types of N-glycan biosynthesis hsa01100 Metabolic pathways	*Low cancer specificity*. *Potential prognostic* ** Kidney Renal Clear Cell Carcinoma *Potential prognostic* * Thyroid Carcinoma
**S100A12**	P80511	Protein S100-A12	hsa04990 EF-hand domain-containing proteins >S100 proteins	*Cancer enriched* Head and Neck Squamous Cell Carcinoma. *Potential prognostic* * Stomach Adenocarcinoma.

**Table 3:** Mean and standard deviation of AUROC values for prediction models using single biomarkers. Values represent the highest mean AUROC (mAUROC) obtained between logistic regression and random forest models for each prediction task. Green highlighting indicates mAUROC >70% for a single biomarker in a specific classification task.

	Healthy vs. Non-Healthy		Gastritis vs. Non-Gastritis		Preneoplasia vs. Non-Preneoplasia		Cancer vs. Non-Cancer		Cancer or Preneoplasia vs. Healthy or Gastritis		All Stages	
	mAUROC	*sd*	mAUROC	*sd*	mAUROC	*sd*	mAUROC	*sd*	mAUROC	*sd*	mAUROC	*sd*
**Age**	**80.1%**	*6.9%*	60.2%	*8.1%*	**71.8%**	*8.8%*	62.2%	*10.0%*	**75.1%**	*8.1%*	63.2%	*4.8%*
**Gender**	57.0%	*10.9%*	57.7%	*7.7%*	49.6%	*2.4%*	66.0%	*10.4%*	55.4%	*9.5%*	56.6%	*4.0%*
***Hp*** **status**	48.2%	*4.9%*	67.2%	*15.3%*	50.9%	*3.3%*	56.8%	*6.1%*	54.5%	*4.8%*	59.6%	*5.0%*
**DCD**	57.4%	*7.0%*	60.5%	*8.2%*	63.7%	*8.7%*	63.6%	*10.2%*	55.7%	*5.9%*	57.1%	*5.0%*
**IGFALS**	63.4%	*8.7%*	59.6%	*8.0%*	62.5%	*9.3%*	58.3%	*8.0%*	61.2%	*7.8%*	54.1%	*4.9%*
**LEP**	**77.0%**	*8.5%*	65.1%	*11.4%*	62.4%	*8.9%*	68.4%	*8.8%*	63.2%	*9.1%*	62.7%	*6.5%*
**KRT14**	63.3%	*7.9%*	**72.3%**	*8.4%*	61.3%	*8.0%*	58.1%	*6.6%*	55.8%	*5.0%*	63.3%	*4.4%*
**MAN2A1**	66.0%	*8.0%*	60.3%	*7.8%*	60.7%	*8.3%*	58.8%	*8.2%*	59.5%	*7.4%*	57.8%	*4.8%*
**KIF20B**	57.7%	*8.2%*	57.4%	*7.7%*	60.4%	*7.6%*	62.1%	*8.8%*	57.4%	*7.9%*	49.9%	*5.7%*
**ARG1**	60.2%	*5.9%*	68.0%	*10.9%*	60.1%	*8.6%*	**75.5%**	*6.3%*	66.1%	*7.0%*	59.7%	*5.9%*
**F13A1**	65.6%	*7.8%*	60.5%	*7.1%*	60.0%	*7.4%*	59.1%	*6.6%*	55.3%	*6.0%*	50.4%	*5.1%*
**S100A12**	57.1%	*8.4%*	59.5%	*8.3%*	59.9%	*8.3%*	61.6%	*10.9%*	58.3%	*6.5%*	53.8%	*5.7%*
**LBP**	**70.7%**	*6.9%*	60.6%	*9.3%*	59.5%	*7.1%*	68.4%	*9.3%*	67.6%	*6.2%*	61.1%	*4.4%*
**ATAD3B**	61.3%	*8.4%*	61.0%	*7.9%*	59.5%	*8.0%*	59.7%	*8.2%*	57.3%	*8.4%*	50.6%	*7.4%*
**JUP**	**71.4%**	*8.1%*	58.6%	*7.7%*	59.3%	*6.8%*	63.2%	*9.9%*	65.2%	*6.6%*	58.8%	*5.4%*
**CA2**	64.9%	*8.6%*	63.9%	*10.8%*	58.8%	*7.8%*	**78.1%**	*8.7%*	69.4%	*7.7%*	62.0%	*6.1%*
**HPT**	62.0%	*7.9%*	61.1%	*8.9%*	58.5%	*6.2%*	64.1%	*10.0%*	64.8%	*8.1%*	55.6%	*4.9%*
**KRT19**	63.3%	*9.5%*	61.0%	*9.3%*	57.7%	*8.5%*	60.4%	*7.7%*	56.0%	*7.2%*	53.3%	*3.8%*
**Max** **mAUROC**	80.1%	*6.9%*	72.3%	*8.4%*	71.8%	*8.8%*	78.1%	*8.7%*	75.1%	*8.1%*	63.3%	*4.4%*
**Min** **mAUROC**	48.2%	*4.9%*	57.4%	*7.7%*	49.6%	*2.4%*	56.8%	*6.1%*	54.5%	*4.8%*	49.9%	*5.7%*
**Marker with** **best** **mAUROC**	**Age**		**KRT14**		**Age**		**CA2**		**Age**		**KRT14**	

**Table 4:** Mean AUROC values and standard deviations for prediction models incorporating multiple biomarkers. For each classification task, the highest performing biomarker combination is highlighted in green, along with its corresponding mAUROC value. This table represents only the highest mAUROC obtained between logistic regression and random forest models for each classification task and number of biomarkers in the models.

Prediction Task	Number of Biomarkers in the Prediction Model											
	1		2		3		4		5		6	
	mAUROC	*sd*	mAUROC	*sd*	mAUROC	*sd*	mAUROC	*sd*	mAUROC	*sd*	mAUROC	*sd*
**Healthy vs.** **non-healthy**	80.1%	*6.9%*	87.7%	*5.0%*	88.0%	*5.3%*	87.4%	*6.9%*	86.2%	*7.9%*	85.2%	*8.2%*
	Age		Age, Hp status		**Age, Hp status, HPT**		Age, Hp status, HPT, KRT19		Age, Gender, Hp status, HPT, KRT19		Age, Gender, Hp status, ARG1, F13A1	
**Gastritis vs.** **non-gastritis**	72.3%	*8.4%*	81.4%	*7.3%*	80.6%	*7.5%*	82.2%	*9.2%*	79.6%	*9.2%*	78.0%	*8.0%*
	KRT14		Hp status, KRT14		Hp status, F13A1, KRT14		**Hp status, ARG1, KRT14, F13A1**		Hp status, ARG1, KRT14, F13A1, KIF20B		Hp status, HPT, KIF20B, ARG1, F13A1, KRT14	
**Preneoplasia vs.** **non-preneoplasia**	71.8%	*8.8%*	75.7%	*8.9%*	74.4%	*10.6%*	73.4%	*10.8%*	72.7%	*7.9%*	70.5%	*11.3%*
	Age		**Age, Gender**		Age, Gender, KRT14		Age, Gender, KRT14, ARG1		Age, Gender, KRT14, KRT19, F13A1		HPT, Leptin, S100A12, KIF20B, ATAD3B, CA2	
**Cancer vs.** **non-cancer**	78.1%	*8.7%*	83.0%	*9.4%*	85.2%	*7.0%*	85.3%	*6.4%*	85.0%	*5.7%*	84.9%	*7.4%*
	CA2		CA2, Leptin		ARG1, CA2, S100A12		**ARG1, CA2, F13A1, S100A12**		Gender, ARG1, CA2, MAN2A1, IGFALS		Age, Gender, ARG1, CA2, MAN2A1, IGFALS	
**Cancer/preneoplasia vs. healthy/gastritis**	75.1%	*8.1%*	76.7%	*7.7%*	78.8%	*6.8%*	81.2%	*6.8%*	83.9%	*5.1%*	81.4%	*6.3%*
	Age		Age, Leptin		Age, HPT, ARG1		ARG1, CA2, HPT, LBP		**ARG1, CA2, HPT, MAN2A1, LBP**		ARG1, CA2, HPT, MAN2A1, LBP, DCD	
**All stages**	63.3%	*4.4%*	71.6%	*5.4%*	74.8%	*4.2%*	76.2%	*6.9%*	77.9%	*7.7%*	75.6%	*6.3%*
	KRT14		Age, Hp status		Age, Gender, Hp status		Age, Hp status, ARG1, KRT14		**Age, Gender, Hp Status, ARG1, KRT14**		Age, Gender, Hp Status, ARG1, KRT14, F13A1	
**Number of tested combinations**	*18*		*153*		*816*		*3060*		*8568*		*18*,*564*	

## Data Availability

The mass spectrometry proteomics data have been deposited to the ProteomeXchange Consortium via the PRIDE partner repository with the dataset identifier PXD062318. To access, log in to the PRIDE website using the following details: Project accession: PXD062318; Token: Shdm5Ynx0pyo. https://www.ebi.ac.uk/pride (accessed on 27 March 25).

## Supplementary Materials

The following supporting information can be downloaded at: https://www.mdpi.com/article/10.3390/ijms262010114/s1. References [67,68,69,70] are mentioned in Supplementary.

## Abbreviations

The following abbreviations are used in this manuscript:

AG	Atrophic Gastritis
AKT	Protein kinase B
AUROC	Area Under the Receiver Operating Characteristics
ATAD3B	ATPase family AAA domain containing 3B
ARG1	Arginase-1
CA2	Carbonic anhydrase 2
CA12-5	Carbohydrate Antigen 12-5
CA19-9	Carbohydrate Antigen 19-9
CAPZA1	Capping Actin Protein of Muscle Z-Line Subunit alpha
CEA	Carcinoembryonic Antigen
DCD	Dermcidin
DDA	Data-Dependent Acquisition
DIA	Data-Independent Acquisition
DYS	Dysplasia
ELISA	Enzyme-Linked Immunosorbent Assays
ERAP2	Endoplasmic Reticulum Aminopeptidase 2
F13A1	Coagulation factor XIII A chain
FC	Fold-Change
FDR	False Discovery Rate
FUCA2	Alpha-L-Fucosidase 1
GC	Gastric Cancer
GI	Gastrointestinal
GPLD1	Glycosylphosphatidylinositol phospholipase D
HPT	Haptoglobin
IGFALS	Insulin-like growth factor-binding protein complex acid labile subunit
IGHG1	Immunoglobulin heavy constant gamma 1
IL1RAP	Interleukin-1 receptor accessory protein
IM	Intestinal Metaplasia
JUP	Junction Plakoglobin
KIF20B	Kinesin-like protein KIF20B
KRT14	Keratin, type I cytoskeletal 14
KRT19	Keratin, type I cytoskeletal 19
LBP	Lipopolysaccharide-binding protein
LC-MS/MS	Liquid Chromatography coupled to tandem Mass Spectrometry
LEP	Leptin
MAN2A1	Alpha-mannosidase 2A1
MAPK	Mitogen-activated protein kinase
mTOR	Mammalian target of rapamycin
PG	Pepsinogen
PI3K	phosphoinositide 3-kinase
PLS-DA	Partiel Least Square-Discriminated Analysis
PRCP	Lysosomal Pro-X carboxypeptidase
PRSS3	Serine protease 3
S100A12	S100-A12 protein
SAA1/2	Serum amyloid A-1 protein/Serum amyloid A-2 protein
STAT3	Signal transducer and activator of transcription 3
SVEP1	Sushi Von Willebrand Factor Type A, EGF &Pentraxin Domain containing 1
TFRC	Transferrin Receptor Protein 1
